# Effects of Aerobic Exercise on Oxidative Stress in Patients Diagnosed with Cancer: A Narrative Review

**DOI:** 10.7759/cureus.5382

**Published:** 2019-08-13

**Authors:** Sara K Arena, Deb J Doherty, Allison Bellford, Gregory Hayman

**Affiliations:** 1 Physical Therapy, Oakland University, Rochester, USA; 2 Human Movement Science, Oakland University, Rochester, USA

**Keywords:** cancer, oncology, neoplasm, oxidative stress, exercise, physical activity

## Abstract

Background: Oxidative stress (OS) can bring about an imbalance between the production of free radicals (pro-oxidants) and their elimination by protective mechanisms (antioxidants). Exercise and/or physical activity (PA) may provide a mechanism to control the variation and equilibrium between pro-oxidants and antioxidants.

Purpose: The purpose of this narrative review is to investigate the evidence regarding the effect of exercise and/or PA on OS among individuals diagnosed with cancer.

Methods: A narrative review study design involved a literature search (August 2016) across the databases: Cumulative Index of Nursing and Allied Health Literature (CINAHL), Cochrane, Excerpta Medica database (Embase), and PubMed. Articles included those published from January 2000 - August 2016; inclusive of the search terms “cancer” AND “neoplasm” AND “oncology” AND “oxidative stress” AND “exercise” AND “physical activity”; written in the English language; and utilizing human subjects. The references of the selected articles were then reviewed to identify any qualifying articles. A modified Strengthening the Reporting of Observational Studies in Epidemiology (STROBE) review of each article was completed by two investigators.

Results: Eight articles met the final inclusion criteria. Moderate exercise may provide protective mechanisms against OS via increased antioxidant activity, while exhaustive exercise may be responsible for increased levels of OS, increasing the risk for malignancy. While increased OS levels are utilized by current oncologic therapies to damage malignant and premalignant cells, they also damage healthy cells (cardiac, nerve, and lymphatic).

Conclusion: Moderate levels of exercise and/or PA may provide preventative and protective qualities against the negative side effects associated with increased OS from cancer treatment.

## Introduction

Oxidative stress (OS) can bring about an imbalance between the production of free radicals and reactive metabolites, so-called oxidants or reactive oxygen species (ROS), and their elimination by protective mechanisms, referred to as antioxidants [1]. Free radicals, commonly referred to in the literature as ROS, are a byproduct of a chemical reaction known as oxidation. Conversely, antioxidants are molecules that inhibit oxidation of other molecules, thereby providing cell protection [1], The body is exposed to OS from both exogenous origins, including air pollution or tobacco smoke, and endogenous origins, such as in mitochondria where oxygen is consumed [2]. An imbalance of ROS and antioxidants in favor of oxidation is characterized by damage to cellular constituents, such as deoxyribonucleic acid, ribonucleic acid, and other structures, which may ultimately cause cell apoptosis or death [3-4]. Evidence suggests an association between this imbalance and both the formation and progression of cancerous cells [2]. Cancer cell production and proliferation is known to increase levels of ROS [1]. They also rely on a simultaneous increase in antioxidant defense for those cells [5]. While the levels of ROS and antioxidant defense in cancer cells is elevated, their tolerance threshold of ROS is lower than normal cells [6]. 

There are numerous mechanisms responsible for the proliferation of malignant cells associated with a cancer diagnosis that are not yet fully understood. Hormesis is one proposed theory which suggests low doses of toxins can have beneficial effects on health and longevity while higher levels would be harmful. The hormesis theory has been extended into a ROS and exercise framework by Radak, Chung, and Goto [7]. They theorized that while a single bout of exercise at certain intensities and duration can increase the production of ROS causing excessive oxidative stress, regularly scheduled exercise at a safe level of intensity and duration can provide beneficial adaptations to body systems, thereby thwarting diseases associated with OS. This concept provides a foundational framework for the molecular basis of preventive effects of regular exercise and the subsequent ability of the body to protect against stronger stresses [7].

Current treatments for cancer, including chemotherapy (CT) and radiation therapy (RT), utilize approaches aimed at targeting malignant cell death. These therapies manipulate the balance within cancer cells by elevating levels of ROS beyond their tolerance, causing cell damage, and ultimately, death in cancerous cells. The resulting and therapeutic physiological effects of these therapies are often an elevation or reduction in ROS outside of the tolerable range for a cancer cell. This ROS-mediated cell death is actually a primary method by which cancer treatments attempt to attack cancerous cells. However, the results of these therapies, even when successful, can have dramatic negative effects on the cardiac, neurologic, and lymphatic systems of the body [8]. A departure from the equilibrium between pro-oxidant and antioxidant systems often results in elevated ROS levels which is termed OS [4]. There is a fine balance between the benefits of OS and the physical damage or adverse effects of OS that can be caused by cancer and its treatments. Therefore, the authors aim to determine if there is evidence to understand and integrate the evidence regarding OS, cancer, cancer treatment, and exercise. This evidence can be used to create physical therapy-focused interventions aimed at primary, secondary, and tertiary prevention.

Evidence suggests that exercise induces OS by increasing both muscular and intracellular activity in response to force and energy demand [9-11]. The degree of benefit or harm in varied types of cancers with varying intensities of exercise requires further examination [12]. Additionally, it is unknown if optimal exercise dosage parameters have been reported that would be of benefit in thwarting cancer cell proliferation or optimizing adjunctive therapies to prevent adverse effects. Physical therapists are well-positioned to utilize this information as they examine, evaluate, and provide exercise prescriptions to individuals with a variety of cancer-related diagnoses. Therefore, the purpose of this narrative review is to investigate the evidence regarding the effect of exercise and/or PA on OS among individuals diagnosed with cancer. 

## Materials and methods

A literature search was conducted in August of 2016 utilizing computerized databases, including the Cumulative Index of Nursing and Allied Health Literature (CINAHL), Cochrane, Excerpta Medica database (Embase), and PubMed. The search terms used for this inquiry included: “cancer” AND “neoplasm” AND “oncology” AND “oxidative stress” AND “exercise” AND “physical activity”. These search terms were organized into three columns for further distinction during the search. The three search term columns were as follows: 1) cancer, neoplasm, oncology; 2) oxidative stress; 3) exercise, physical activity. Articles were included if they were published between January 2000 and August 2016, found in full text in peer-reviewed journals, studied human subjects, were written in the English language, and contained information in each of the three search columns. Articles that were duplicates or that did not meet these criteria were excluded.

While it is widely accepted that exercise and/or physical activity (PA) provides health benefits to patients diagnosed with cancer throughout the continuum of care, its relationship to OS, cell malignancy, or as an adjunctive to cancer treatments has only limited evidence available to those prescribing exercise, including physical therapists (PTs) [9-10]. The terms "exercise" and "PA" are used interchangeably by the authors of this narrative to describe similar concepts of bodily movement and aerobic capacity, as the terms described similar concepts within the articles reviewed.

**Figure 1:**
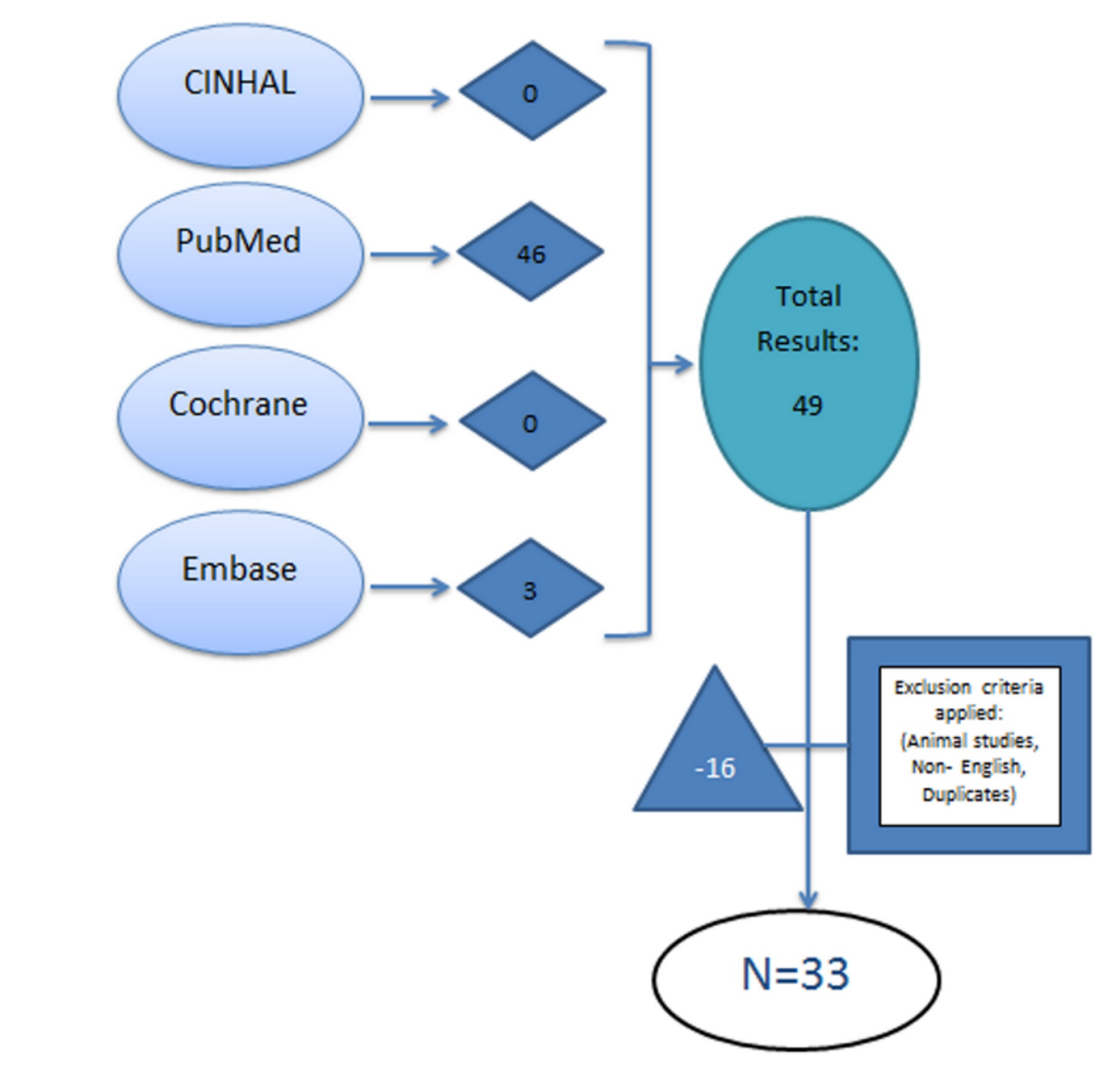
Literature search with exclusion criteria applied CINAHL: Cumulative Index of Nursing and Allied Health Literature; Embase: Excerpta Medica database

**Figure 2:**
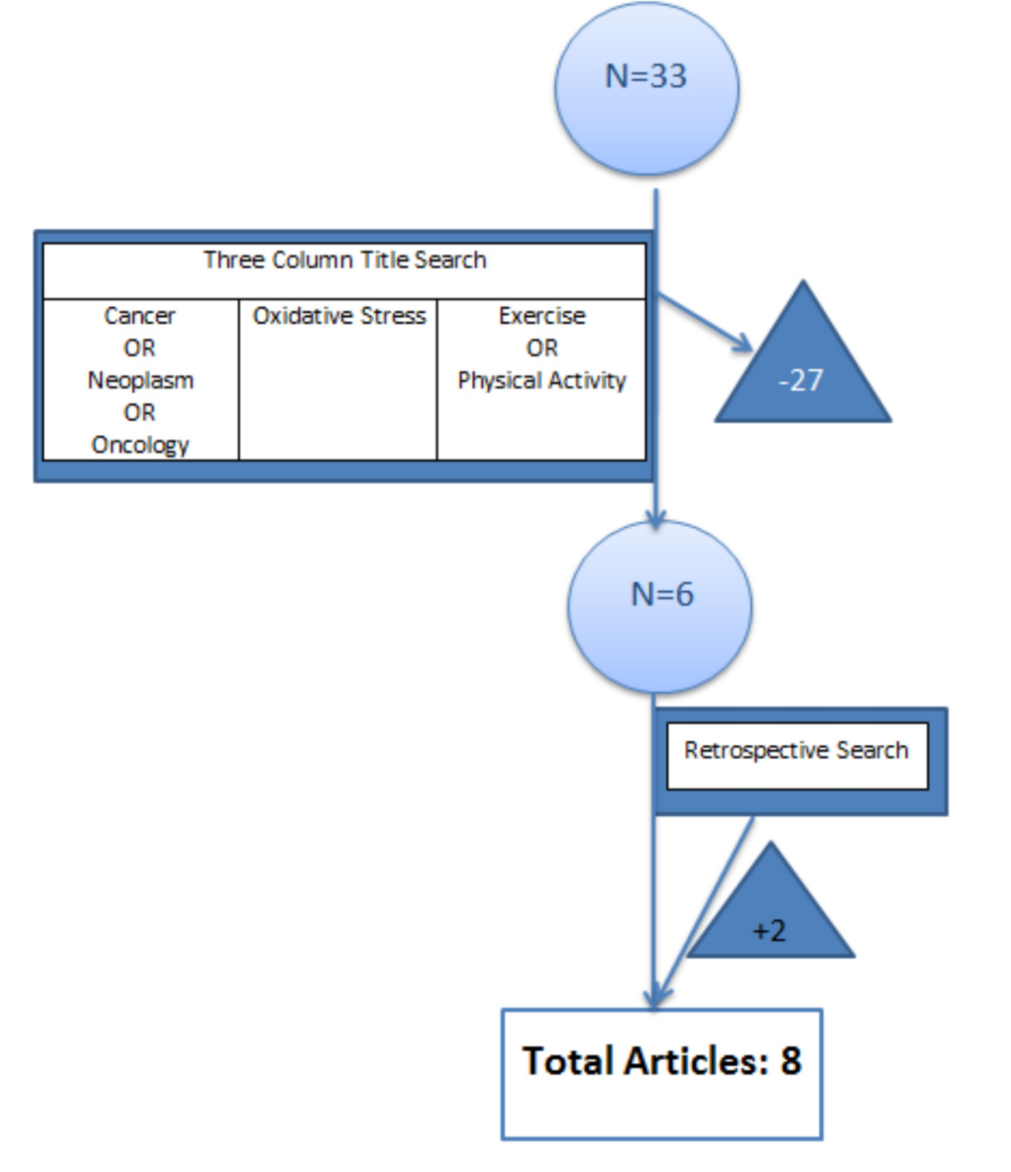
Three column title search applied followed by retrospective search

Forty-nine articles were generated prior to the application of inclusion or exclusion criteria (Figure 1). Sixteen articles were excluded for the use of animal data (n = 10), duplicate articles (n = 5), or written in non-English with no available translation (n = 1). A three-column title search criterion was then utilized. Each article chosen had to discuss cancer, OS, and exercise or PA. Upon utilizing this criterion, 27 more articles were eliminated, resulting in a total of six articles. A retrospective search of the references of the six articles was performed utilizing the same criteria. This yielded an additional two articles, bringing the total to eight (Figure 2). 

Data were extracted using 11 criteria: type of research/study design; definitions of OS; OS and its link to cancer; OS and its link to PA; type/location of cancer; stage of cancer; dosage of activity reported; reference to physical therapy; testing for OS; mortality; and study limitations. Articles were further evaluated for information about epigenetic factors, such as smoking history, sleep habits, stress, body mass index (BMI), and nutrition. Finally, documented use of primary, secondary, or tertiary prevention activities was evaluated. In the context of cancer, primary prevention entails intervening before the onset of malignant cell proliferation. Secondary prevention refers to reducing the impact of adverse side effects brought about by cancer or its associated treatments, as well as screening to identify cancers in the earliest stages and before the onset of signs and symptoms. Tertiary prevention refers to managing adverse side effects caused by cancer treatments, as well as managing the disease post-diagnosis, during, and after treatment to slow, stop, or manage disease progression [13].

A Strengthening the Reporting of Observational Studies in Epidemiology (STROBE) analysis was performed on all eight articles. While the investigators recognize the STROBE is primarily utilized for observational studies, the use of one analysis tool for review of all articles was used to prevent review bias and provide consistent outcomes for comparison. Modifications to the tool in regard to the type of data extracted during the article review were made to assure quality, minimize bias, and garner additional data inclusive of reported age, sex, and epigenetic factors. Each article was independently evaluated by a minimum of two investigators; the areas of concern were then discussed to come to a consensus.

## Results

Of the eight articles that met inclusion criteria, four articles were narrative reviews, two were pilot studies, one case-control, and one randomized control trial. Cancer diagnoses within these articles included lung [6, 14-15], breast [3, 16-17], prostate [17-18], and colorectal [17, 19]. Only the studies involving lung cancer specified the stage, two of which were Stage I-IIIb NSCLC [6, 14] and one Stage I-IIIA non-small cell lung carcinoma (NSCLC) [15]. None of the studies gave information regarding the mortality of their participants. Table 1 provides demographics related to each inclusion article.

**Table 1:** Summary of Article Findings CA: carcinoma; CI: confidence interval; DNA: deoxyribonucleic acid; HI: high intensity; MI: moderate intensity; OR: odds ratio; OS: oxidative stress; PA: physical activity; ROS: reactive oxidative stress; TNF: tumor necrosis factor ♀: female; ♂: male; 1°: primary prevention; 2°: secondary prevention; 3°​​​​​​​: tertiary prevention

Author, publication year	Study design	Sex of participants	Age of participants (in years)	Location of cancer diagnosis	Findings supporting positive effects of OS with exercise/PA	Findings supporting negative effects of OS with exercise/PA	Exercise as CA prevention reported
Filaire et al., 2013 [6]	Narrative review	♀ and ♂	Not available	Lung	MI PA reduces OS and future tumor development risk	Exhausting PA increases DNA damage and may increase cancer risk which may be related to the degree of OS	1°, 2°, 3°
Gago-Dominguez et al., 2007 [16]	Narrative review	Not available	Not available	Breast	Physical exercise can induce OS and subsequent apoptosis of pre-malignant and malignant cells protecting against breast CA	Lymphocyte counts and function decreases with exhaustive exercise	1°
Rebillard et al., 2013 [18]	Narrative review	Not available	Not available	Prostate	PA has a protective effect from prostate cancer; MI exercise reduces ROS	Exhaustive exercise increases ROS	1°​​​​​​​
McTiernan, 2008 [17]	Narrative review	Not available	Not available	Breast, colon, prostate	PA might reduce systemic inflammation alone or in combination with a reduction in body weight or change in body composition	Not available	1°​​​​​​​, 2°, 3°​​​​​​​
Jones et al., 2009 [14]	Pilot study	7 ♀ 5♂	Mean 67 ± 8	Lung	All systemic inflammatory markers except TNF-a were lower following exercise training	Not available	2°​​​​​​​, 3°​​​​​​​
Jones et al., 2011 [15]	Pilot study	8 ♀, 8 ♂	Mean 63 ± 10	Lung	Not available	Chronic aerobic training was associated with a significant increase in biomarkers of OS	2°​​​​​​​, 3°​​​​​​​
McCullough et al., 2012 [3]	Case-control	♀ 1,053 cases and 1,102 controls	Range 20 - 98	Breast	A 30% risk reduction was observed in the third quartile of PA (OR = 0.70; 95% CI, 0.52 - 0.95)	Highest quartile of PA experienced a 16% risk reduction (OR = 0.84, 95% CI, 0.63 - 1.13)	1°​​​​​​​
Allgayer et al., 2008 [19]	Randomized control trial	17 in MI groups 12♂/5♀ 27 in HI group 15♂/12♀	58 ± 3 in MI; 59 ± 1 in HI	Colon	MI exercise decreases OS	HI exercise increases OS	2°​​​​​​​, 3°​​​​​​​

A definition of PA was described by Filare et al. as leisure-time PA (LPA) which referred to short-term, intensive energy expenditure and occupational PA (OPA), which occurs over longer periods (e.g., hours) at lower rates of energy expenditure [6]. Furthermore, exhaustive exercise was referred to as 80% of VO_2_ max, while moderate exercise refers to 60% of VO_2_ max in the article by McCullough et al. [3]. It is notable that Gago-Dominguez et al. suggested the specific dosage of PA necessary to infer a reduction in cancer risk was not fully understood but indicated three to four or more hours per week may be necessary for risk reduction [16].

There are varied ways to measure OS. The most common OS testing methods employed measurement of F2-isoprostanes, 8-oxo-dG, and other pro-inflammatory markers in urine [6, 14, 16-18]. However, Jones et al. measured the intercellular adhesion molecule 1 (ICAM-1), macrophage inflammatory protein 1-alpha (MIP-1α), interleukin-6 (IL-6), interleukin-8 (IL-8), monocyte chemotactic protein-1 (MCP-1), and tumor necrosis factor-alpha (TNF-α) in blood plasma to obtain this measurement [15]. Alternatively, McCullough et al. used 10 functional polymorphisms from nine genes in the OS pathway: CAT (rs 1001179), COMT (rs4680 and rs737865), GPX (rs 1050450), GST AI (rs3957356), GSTM1 (gene deletion), GSTP1 (rsl695), GSTT1 (gene deletion), MnSOD (rs4880), and MPC (rs2333227) using genotyping. Blood samples were provided by participants for measurement [3]. In summary, the lack of one consistent testing methods limits direct comparisons of the oxidative damage and antioxidant reserve observed in each study.

In respect to OS and its relationship to primary cancer, six articles noted that high levels of OS cause damage in DNA and human tissue and could increase the risk of future malignancy [3, 6, 15-16, 18-19]. Filaire et al., however, also suggested that high levels of OS caused apoptosis and damage to premalignant and malignant cells, acting as a protective mechanism against both developing and spreading cancer [6]. Table 1 provides detail of this observation

Oxidative stress was further linked to PA, exhibiting a delicate relationship. Four studies described how moderate PA alleviates OS damage via the expression of antioxidants [3, 6, 18-19]. However, six studies noted that exhaustive PA increases levels of OS and results in increased DNA and tissue damage [3, 6, 15-16, 18-19]. Table 1 provides specifics related to each article.

Five articles included primary prevention strategies [3, 6, 16-18], five included secondary prevention strategies [3, 14-15, 17, 19], and five included tertiary prevention strategies [3, 14-15, 17, 19]. Primary prevention related to smoking [6, 14-15, 19] and/or nutrition [6, 17-19] were mentioned in a total of six articles. None of the studies in this review included a discussion of physical therapy-specific intervention. Table 1 provides additional specifics related to prevention level discussion from each article.

## Discussion

The purpose of this review was to investigate the evidence regarding the effect of exercise and/or PA on OS levels among individuals diagnosed with cancer. Levels of OS within articles identified in this narrative review were measured in varying ways, including urinary markers, blood testing, and genotyping. Furthermore, the OS measurement techniques employed within each type of cancer or diagnostic category differed even further. This supports previous reports suggesting that measures of oxidative status are variable and susceptible to significant technique differences across laboratories [20] which impedes the comparisons among the articles used in this narrative review. Further research with an intention toward consistency in OS marker utilization (based on evidence of which marker would provide the most valid and reliable measures of change related to exercise in those with cancer) is warranted for broader outcome interpretation. 

The analysis of the articles meeting the inclusion criteria exposed inconsistencies in the terminology and application of exercise and PA in regards to types, dosages, and intensities (i.e., LPA, OPA). These inconsistencies may stem from a non-uniform and changing definition of exercise and PA guidelines [21]. In 2012, the American College of Sports Medicine convened a multidisciplinary group of experts who reviewed the evidence and developed guidelines for PA for cancer survivors which have added more understanding and parameters for exercise [22]. The differing levels of intensity in PA yielded varying conclusions regarding the effects of exercise on OS levels. This made it difficult to suggest the most effective and safe level of exercise when prescribing exercise to individuals with cancer. While this review revealed some evidence for moderate PA enhancing the antioxidant defense systems to protect against oxidative damage [15, 17-19], it was confounded by other findings suggesting PA at exhaustive levels stretched beyond these protective capabilities, thereby increasing OS to damaging levels [3, 6, 14, 16, 19]. Furthermore, this review found that variable exercise intensities had differing effects on the balance of the pro- and antioxidant balance in the body and that OS levels play varying roles in the development, proliferation, and treatment of cancer. Increased OS levels cause damage to DNA, tissues, etc. and place patients at greater risk for cancer [3, 18-19]. These findings are in agreement with previous reports suggesting OS can cause inflammation that may serve as the impetus to cancer formation; however, it may also be beneficial as an adjunctive cancer treatment to damage cancer cells [1]. Simultaneously, while it may potentially kill cancer cells, OS can damage healthy cells and cause adverse side effects to organs, such as the heart, brain, lymphatic system, and peripheral nerves. This reaffirms the importance of maintaining the pro- and antioxidant balance for cancer prevention and cancer-related interventions. Notably, CT and RT are therapies utilized to exploit this imbalance through targeted and non-targeted increases in levels of OS, to which cancerous cells are more sensitive, in order to initiate cancer cell apoptosis [23]. 

A hypothesized mechanism for the benefits of exercise for individuals diagnosed with cancer utilizes the process of hormesis, in which repeated low doses of an otherwise damaging substance increase tolerance levels [15]. With regards to cancer and exercise, this would be a primary or secondary prevention approach in which low to moderate amounts of OS could be applied to the body using exercise dosed at a beneficial low to moderate level of intensity [15, 17-19]. This would aid in increasing the threshold for quantities of OS that can be applied to normal cell’s antioxidant defense system before damage occurs. Therefore, it is possible that by utilizing this principle, the body would be able to handle higher levels of OS during therapies, such as CT or RT, before damage occurs to normal cells. In other words, a moderately dosed exercise prescription provided and performed by individuals throughout the course of CT and/or RT interventions should be considered as it could provide a protective mechanism for normal cells. The use of repetitive temporary OS initiated through exercise could aid in increasing the threshold to cellular damage and side effects of cancer therapies [4]. An increased threshold to damage could lead to the potential for increased cancer treatment completion rates through a reduction in symptoms, such as fatigue, in which OS plays a role [4, 23-24].

Furthermore, these findings suggest that aerobic exercise at high to exhaustive intensities can overwhelm the antioxidant defense system and cause oxidative damage [3, 6, 14, 16, 19]. Conversely, moderate aerobic exercise activity appeared to have the opposite effect by enhancing the antioxidant defense system [15, 17-19]. Clinically, these temporary increases in OS could push cancer cells outside of their protective threshold, leading to apoptosis [6].

In summary, there is evidence regarding the positive benefits of exercise or PA to the promotion of health status, as well as management and prevention of disease, including cancer [22, 25-26]. However, conclusive evidence related to the OS-PA relationship in individuals with a cancer diagnosis and undergoing cancer-related treatments will require further examination. Moderate exercise has been shown to improve the control of OS in the human body [15, 17-19]. Therefore, control of OS levels through exercise prescription could potentially aid in treatment completion, symptom reduction, and limit the quality of life for those diagnosed with cancer. This study suggests that exercise or PA could be a valuable part of primary, secondary, and tertiary prevention approaches among persons at risk for cancer and throughout the continuum of cancer care. Exercise prescription is one of the major areas in the scope of practice for PTs who are very well-positioned to provide primary prevention through public health education and individual or group exercise programs, secondary prevention through pre-habilitative services or patient-centered exercise programming for those with a cancer diagnosis, and tertiary prevention through optimizing functional status and minimizing adverse effects for those undergoing cancer treatments. 

Study limitations

Study limitations include a small number of articles that met the inclusion criteria, small sample sizes in the aforementioned articles, the lack of a non-exercise control group limits comparison, lack of randomized controlled trials, and a wide variability in the protocols, exercise program design, how OS was measured, and the duration of intervention periods. Additionally, studies meeting the inclusion criteria did not encompass methodologies that examined pre- and post-changes in the amount of ROS and antioxidants resulting from exercise participation, thereby limiting the investigators' ability to fully address the study purpose. Furthermore, the search methodology of this review could have resulted in more articles for inclusion if search terms that allowed OR between similar search terms, as well as AND between each of the three topics, were used. Finally, as this study was a narrative review and not a systematic review or meta-analysis, reporting of findings using a Preferred Reporting Items for Systematic reviews and Meta-Analyses (PRISMA) tool was not utilized.

Future research

This topic would benefit from future novel research by designing randomized controlled trials, standardizing definitions and measures for OS, exercise intensity, or protocol, and how primary, secondary, and tertiary outcomes are assessed. Additionally, longitudinal studies throughout the continuum of cancer care with efforts toward measures of OS and prescriptive exercise dosing are needed to build firm support for exercise and the associated safe amounts across age, sex, cancer type, cancer stage, and during various adjunctive treatments for cancer. Finally, an examination of OS differences in sedentary individuals versus highly trained athletes may be helpful to determine if athletes are less impacted by high-intensity exercise and the associated OS changes due to physiologic tolerance.

The information gathered during this review may be useful to PTs when providing care to the general public, individuals with an increased risk for developing cancer, and among individuals that already have a cancer diagnosis. The benefits of these findings to individuals receiving physical therapy interventions may rest in the conceptual framework of hormesis, as previously described, or in the constructs of the antioxidant defense system which can be enhanced with the appropriate intensity of exercise. PTs may play a vital role in the development, validation, and application of these concepts into the care plans of relevant patient populations; however, further research is warranted to examine this concept further. 

## Conclusions

Exercise, cancer, and OS have a link that is clinically relevant and requires further investigation to determine the potential exploitation of this relationship for the beneficial treatment of cancer across the continuum of care. Further, exercise may play a role in the primary, secondary, and tertiary preventions across the cancer continuum as a technique to manage OS levels prior to, during, and after cancer treatment. A firm understanding of these mechanisms will aid PTs in prescribing exercise to individuals with a cancer diagnosis and in designing an exercise program of optimal benefit to their current and future health.
